# Effect of Temperature on the Growth of Silver Nanoparticles Using Plasmon-Mediated Method under the Irradiation of Green LEDs

**DOI:** 10.3390/ma7127781

**Published:** 2014-12-05

**Authors:** Shan-Wei Lee, Shi-Hise Chang, Yen-Shang Lai, Chang-Cheng Lin, Chin-Min Tsai, Yao-Chang Lee, Jui-Chang Chen, Cheng-Liang Huang

**Affiliations:** Department of Applied Chemistry, National Chiayi University, Chiayi City 600, Taiwan; E-Mails: s0980272@mail.ncyu.edu.tw (S.-W.L.); s0982723@mail.ncyu.edu.tw (S.-H.C.); s0972207@mail.ncyu.edu.tw (Y.-S.L.); s0980268@mail.ncyu.edu.tw (C.-C.L.); s0972234@mail.ncyu.edu.tw (C.-M.T.); jump_lyc@hotmail.com (Y.-C.L.)

**Keywords:** plasmon-mediated, silver nanoplates, silver nanodecahedra, SERS

## Abstract

Plasmon-mediated shape conversion of spherical silver nanoparticles (NPs) to nanostructures with other shapes under the irradiation of green LEDs (520 ± 20 nm, 35 mw/cm^2^) at various temperatures (60, 40, 20, 10, 5, and 0 °C) was performed in this study. It was found that the bath temperature used in the reaction can influence the reaction rates, *i.e.*, the times needed for the shape transformation process were 5, 11.5, 25, 45, 72, and 100 h at 60, 40, 20, 10, 5, and 0 °C, respectively. In addition, the bath temperature can also alter the morphologies of the final products. The major products are silver nanoplates at 60, 40 and 20 °C. However, they became decahedral silver NPs at 5 and 0 °C. The percentages of decahedral silver NPs synthesized at 60, 40, 20, 10, 5, and 0 °C are 0%, 1%, 5%, 45%, 73%, and 89%, respectively. Measuring the surface-enhanced Raman spectroscopy (SERS) spectra of the probe molecule R6G in the presence of KBr showed that both silver nanoplate colloids synthesized at 60 °C and decahedral silver NP colloids synthesized at 0 °C in the absence of PVP had good SERS activities.

## 1. Introduction

Silver nanoparticles (NPs) have been widely applied to chemical and biological sensors [1,2,3,4], optical devices [5], optical labeling [6] and surface-enhanced Raman spectroscopy (SERS) [7,8,9,10,11,12,13,14,15,16,17,18]. These applications generally originate from the phenomenon of surface plasmon resonances (SPR), which are the collective oscillations of surface electrons induced by the incident light. The frequencies of SPR bands of silver nanoparticles strongly depend on the size, shape, refractive index of the surrounding medium, and the inter-particle distance [19]. It is believed that controlling the shape of NPs is one of the most efficient methods to obtain silver NPs with the desired SPR wavelength [20]. Recently, several groups discovered that silver nanoplates can be tailored by various methods to tune the dipolar SPR wavelength from *ca.* 700 nm to *ca.* 420 nm [21,22,23].

Numerous methods have been developed successfully to synthesize silver nanoparticles with particular shapes, such as tetrahedral [24], bipyramids [25], cubes [26], disks [27,28,29,30,31], decahedra [32,33,34,35,36,37], hexagons [38], triangular plates [39,40,41,42,43,44,45], octahedrons [46], rods [47,48] and wires [49,50]. These methods to synthesize silver NP colloids can be mainly classified into two major categories: direct chemical reduction (thermal) and photochemical approaches. The photochemical approach provides a better way to study the mechanism than thermal reduction methods because these reactions can be terminated immediately by removal of the light irradiation. The absorption of light by the silver nanoparticles can generate a “hot electron” to reduce the silver ions and a “hot hole” to oxidize the citrate ions on the surface and then undergo the shape transformation [51]. It is conceivable that the reaction rates or even the branching ratios can be regulated by varying the reaction temperatures in the thermal reduction reactions. However, very little data has been reported concerning the effect of temperature on the synthesis of silver NPs using photochemical reactions [37,52]. This is possibly because the range of temperature, which can be adjusted in photochemical reactions, normally in aqueous solutions, is limited between 0 °C to 10 °C. Consequently, one would not expect the temperature to cause any significant difference in the reactions over such a narrow range of reaction temperatures.

Light emitting diodes (LEDs) have properties of relatively narrow-band, high emission-intensity and small divergent-angle, so they have been frequently used as the source of excitation light in photochemical reactions to synthesize particular shapes of silver NPs Several methods have been demonstrated in the synthesis of decahedral silver NPs by plasmon-mediated shape conversion from silver spherical NP seeds under irradiation of blue LEDs. [35,37,52,53,54] Additionally, nanodecahedra seeds synthesized in this manner can be further enlarged by the application of green (or other) light via LEDs to tune the Local Surface Plasmon Resonance (LSPR). [54] However, to the best of our knowledge, no results have been reported yet to synthesize high-yield silver nanodecahedra using green LEDs (or other green light sources) directly. Although Xu *et al.* and Scaiano *et al.* [35,52] employed different light wavelengths to control the morphology of NPs, the major products of the plasmon-mediated process under irradiation with green lights in their experimental conditions were silver nanoplates only.

In this study, we synthesized silver NPs by a plasmon-mediated shape conversion approach under irradiation of green LEDs at various temperatures. After systematically varying the reaction temperature from 0 °C to 60 °C, we found that the major products in the reaction were decahedral NPs or nanoplates if the temperature was set lower than 10 °C or higher than 20 °C, respectively. Intuitively, we assumed that the nanodecahedra, which have the larger volume or smaller surface to volume ratio, would have the smaller heat of formation and smaller entropy than the nanoplates. Consequently, the photochemical reaction favors nanodecahedra at a lower bath temperature, but favors nanoplates at a higher bath temperature. Nanodecahedra synthesized at 0 °C are not stable and their corners and edges become round after removing the light source for 2 h under ambient temperature in the presence or absence of light. In contrast, the silver nanoplates are quite stable and can maintain their shape unchanged for more than several months. Both the as-prepared silver nanodecahedra (even the corners and edges became round) and silver nanoplates exhibited very good enhancement ability in measuring SERS spectra of the probe molecule R6G. More remarkably, R6G in silver nanodecahedral colloids synthesized in the presence of Polyvinylpyrrolidone (PVP) and arginine using the method developed by Kitaev *et al.* only showed a very strong fluorescence background. We believed that PVP would hinder the adsorption of R6G onto the surfaces of silver NPs. Therefore, our as-prepared silver NPs had a relatively approachable surface and less limitation in applications of SERS measurements.

## 2. Results and Discussion

### 2.1. Effect of Temperature on the Morphology of Silver Nanoparticles

**Figure 1:**
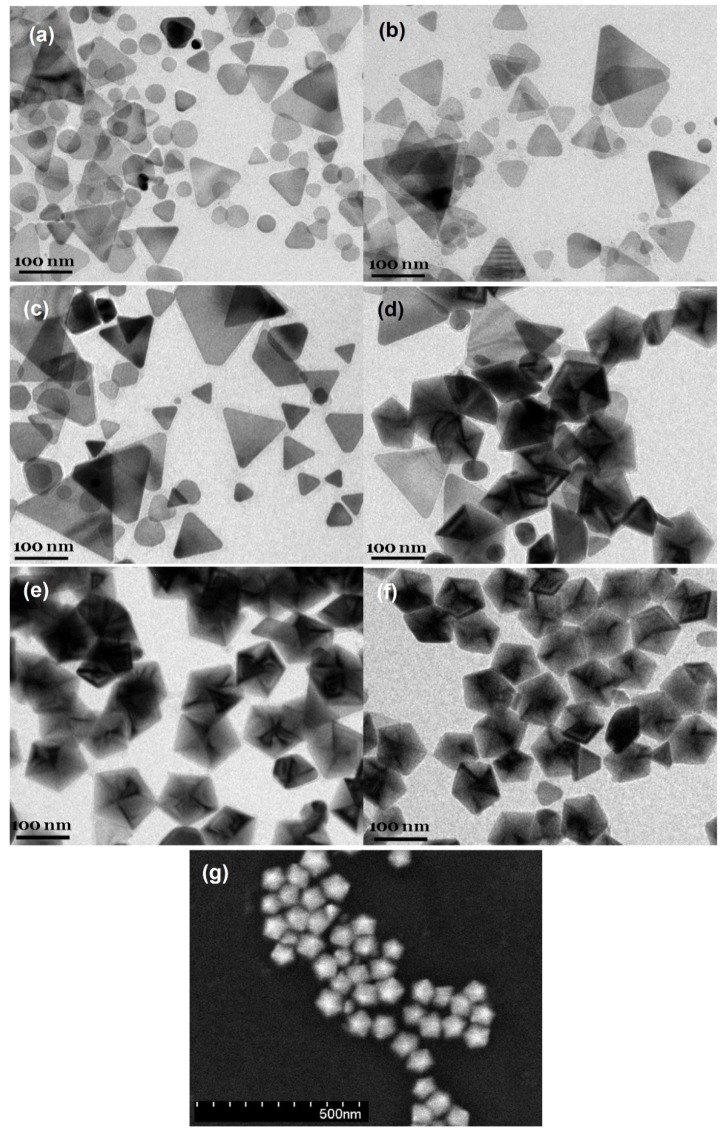
Transmission electron microscopy (TEM) images of the silver nanostructures synthesized at various temperatures: (**a**) 60 °C; (**b**) 40 °C; (**c**) 20 °C; (**d**) 10 °C; (**e**) 5 °C; (**f**) 0 °C and (**g**) the scanning electron microscopy (SEM) images of the silver nanostructures synthesized at 0 °C. The reaction times for 60 °C, 40 °C, 20 °C, 10 °C, 5 °C and 0 °C are 5, 11.5, 25, 45, 72, and 100 h, respectively.

Figure 1a–f shows the transmission electron microscopy (TEM) images of silver nanoparticles synthesized at 0, 5, 10, 20, 40 and 60 °C. The photochemical reactions continued over a long period of time but became very slow at the late reaction stages. Reaction rates were dependent on the bath temperatures and reaction times were chosen when the peak intensities of the dipolar SPR bands in time-dependent spectra reached the maxima. The reaction times needed for the SPR bands to reach the maxima at 60, 40, 20, 10, 5, and 0 °C are 5, 11.5, 25, 45, 72, and 100 h, respectively.

The concentration of sodium citrate, 3 × 10^−3^ M, ten times more concentrated than the previous study [37], was used for the purpose of easily reproducing the results. Because the as-prepared silver nanostructures might not be stable at ambient conditions, A 100 μL sample of 10^−4^ M 16-mercaptohexadecanoic (16-MHDA) was added to the silver colloids prior to the TEM measurement. In our previous studies [21], 16-MHDA formed a self-assembly monolayer on the nanoparticle surface and therefore protects the surface from dissolution and avoids shape transformation. The TEM images show that the major products synthesized at 0 and 5 °C are nanodecahedra, while they become silver nanoprisms or nanoplates at 20, 40, and 60 °C. These results indicate that the factor of temperature not only influences the reaction rate but also affects the morphologies of silver NPs in the plasmon-mediated photochemical reaction. Figure 1g show the SEM images of silver nanoparticles synthesized at 0 °C. The SEM image shows that the size distribution of the silver nanodecahedra prepared at 0 °C is relatively narrow with an average edge length of 65 nm.

**Figure 2:**
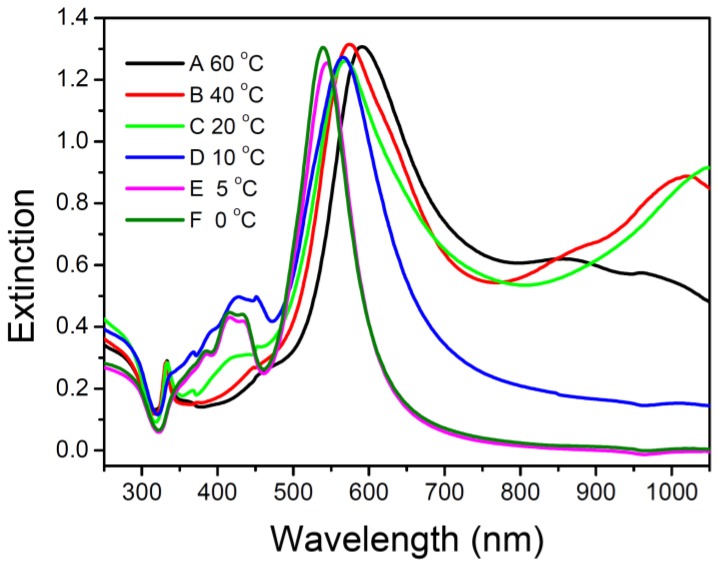
Extinction spectra of silver colloids synthesized at (**A**) 60 °C; (**B**) 40 °C; (**C**) 20 °C; (**D**) 10 °C; (**E**) 5 °C; and (**F**) 0 °C; respectively.

Spectra A-F in Figure 2 show the extinction spectra of the silver nanoparticle colloids synthesized at 60, 40, 20, 10, 5, and 0 °C, corresponding to the TEM images in Figure 1a–f. The major SPR bands in the spectra A, B, C, D, E, and F peak are approximately at 592, 575, 569, 566, 545, and 539 nm, respectively. The peaks at 539, and 545 nm in spectra F and E are attributed to the longitudinal SPR bands of silver nanodecahedra. The SPR wavelength of silver nanodecahedra synthesized at 5 °C is longer than that synthesized at 0 °C. This spectral observation was consistent with the TEM observation that the average edge length with the standard deviation for silver nanodecahedra synthesized at 5 °C is 75.6 ± 9.2 nm, which is longer than that synthesized at 0 °C (average edge length with the standard deviation is 58.4 ± 7.4 nm). The peaks at 592, 575 and 569 nm in spectra A, B and C in Figure 2 correspond to the in-plane dipolar SPR modes of silver nanoprisms or nanoplates. Spectra A, B, and C show small but sharp peaks around 330 nm. The peak at around 330 nm, which is of distinctive character in the optical spectra of the silver nanoplates, corresponds to the out-of-plane quadruple mode of silver nanoplates. In contrast, almost no peak at 330 nm can be observed in the spectra D, E, and F. These results indicate the fact that few products synthesized at 0, 5, and 10 °C are silver nanoplates, which is consistent with the observations in Figure 1d–f. In addition, Spectra A, B, and C show intense peaks at around 900–1050 nm, which are attributed to the in-plane dipolar SPR modes of silver nanoprisms with edge length longer than 100 nm. According to the literature [39,40], these large silver nanoprisms could possibly form from photo-induced fusion of smaller silver nanoprisms or could possibly grow gradually from the smaller nanoplates via the plasmon-mediated process originating from the interaction between in-plane quadruple SPR modes and the excitation light.

**Figure 3:**
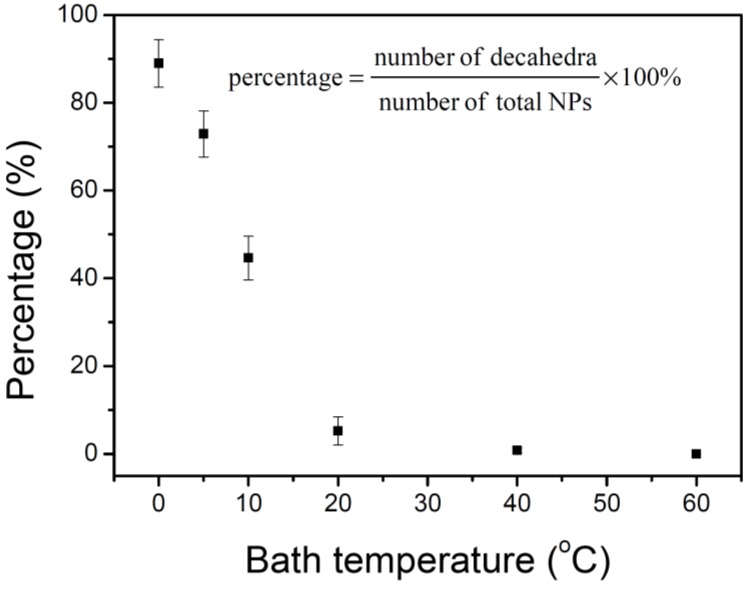
Percentages of decahedral silver nanoparticles in the final products synthesized at various temperatures.

Figure 3 shows the percentages of silver nanodecahedra at different temperatures. The percentage (R) of nanodecahedra is defined as in Equation (1):

(1) $$\text{R}=\frac{N_{deca}}{N_{deca}+N_{tetra}+N_{plate}+N_{other}}\times 100\%$$

where *N_deca_*, *N_tetra_*, *N_plate_*, and *N_other_* represent the number of decahedra, tetrahedrons, plates, and silver nanoparticles with other shapes, respectively. Each data point in Figure 3 was estimated from more than 500 nanoparticles in three independent experiments. It shows that the percentages of nanodecahedra decrease as the bath temperature increases. Because the silver nanostructures might undergo shape transformation under the irradiation of light and the products ratios of these NPs would change in different reaction processes, it is necessary to record the time-dependent extinction spectra and TEM images to follow the crystal growth process.

### 2.2. The Mechanistic Study of Crystal Growth

**Figure 4:**
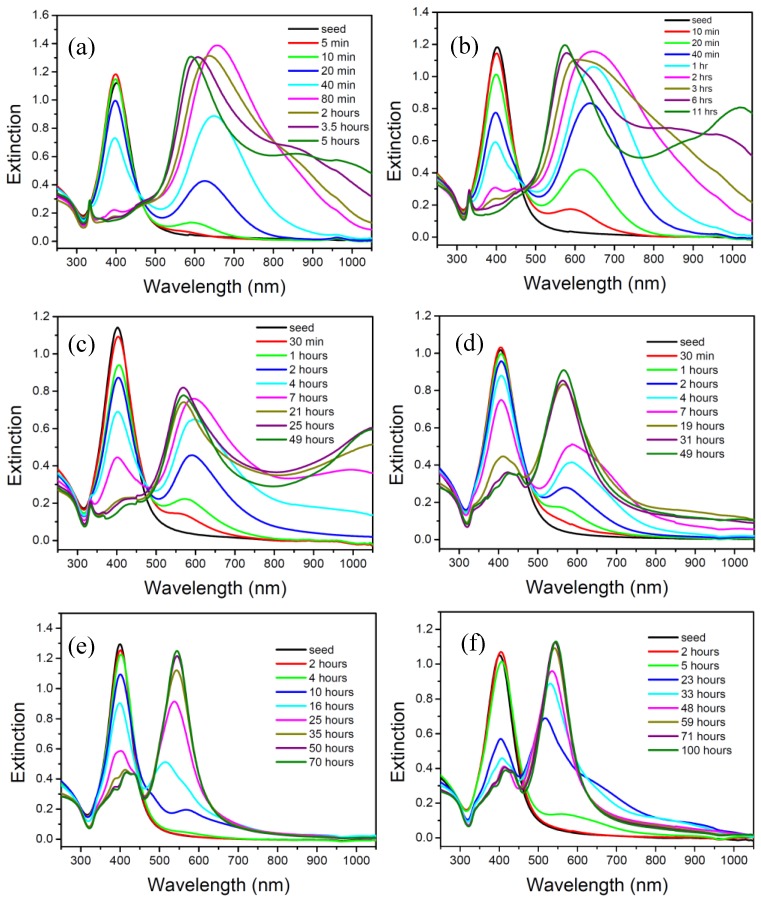
Time-dependent extinction spectra of silver colloids synthesized at various temperatures: (**a**) 60 °C; (**b**) 40 °C; (**c**) 20 °C; (**d**) 10 °C; (**e**) 5 °C; (**f**) 0 °C.

Figure 4a–f show the time dependent extinction spectra of the silver NP colloids synthesized at 60, 40, 20, 10, 5, and 0 °C. Figure 4f shows that the intensity of the peak at 400 nm decreased and the intensity of the peak at 539 nm increased gradually with respect to the reaction time. This observation indicates that the spherical silver NPs transformed into silver nanodecahedra slowly via the plasmon-mediated photochemical process.

**Figure 5:**
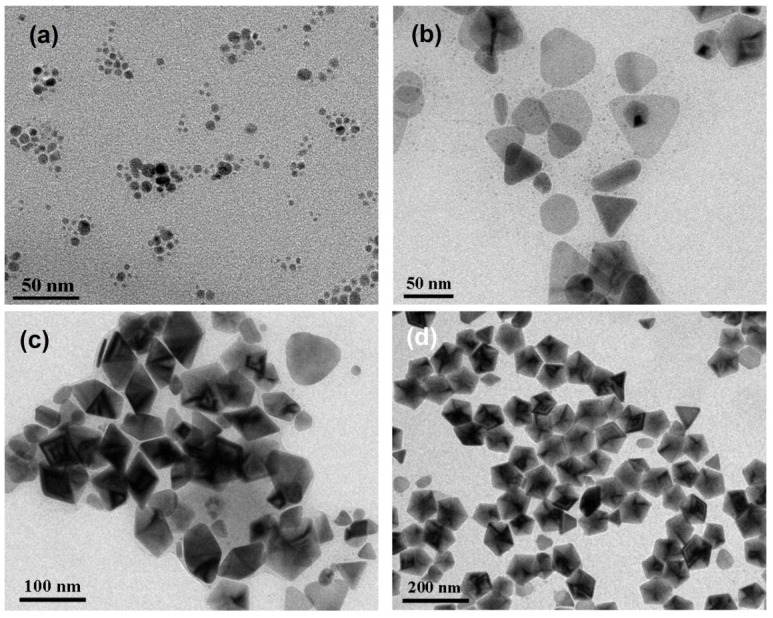
TEM images of the silver nanoparticles synthesized at 0 °C and at different reaction times: (**a**) 1.5 h; (**b**) 23 h; (**c**) 48 h and (**d**) 100 h.

Figure 5a–d show the TEM images of the silver NPs synthesized at 1.5, 23, 48, and 100 h, respectively. At the very beginning of the reaction stage, most of the nanostructures are quasi-spherical NPs, as shown in Figure 5a. After 20 h, many nanoplates can be observed. In the later stage of the photochemical reaction, the nanodecahedra became larger and the number of nanoplates decreased. This observation indicates that the shape evolution of the silver nanostructures followed the process of Ostwald ripening, *i.e.*, the nanoplates sacrificed and provided the silver element source to nanodecahedra, under this experimental condition. At this experimental condition of such a low temperature, the silver nanoplates can be regarded as intermediates, which form at the early reaction stage and almost disappear completely at the final reaction stage.

In contrast, time-dependent spectra of the silver colloids synthesized at 60 °C (Figure 4a) show that the spherical nanoparticles transformed into nanoplates within 80 min. After 80 min, the peak at about 680 nm gradually split into three peaks. Eventually, the major peak blue-shifted from 680 nm to about 590 nm, and two small peaks emerged at around 880 nm and 960 nm. The red-shift of the peaks at around 880 nm and 960 nm is probably attributed to the increasing size of the nanoplates, and the blue-shift is probably attributed to the decreasing size or corner etching of the smaller nanoplates. On the other hand, the sharp peak at 330 nm hardly changed its position at the later reaction stage. This observation indicates that the peak position of the out-of-plane quadruple SPR band would not be very sensitive to the evolution of the sizes and shapes of the nanoplates.

**Figure 6:**
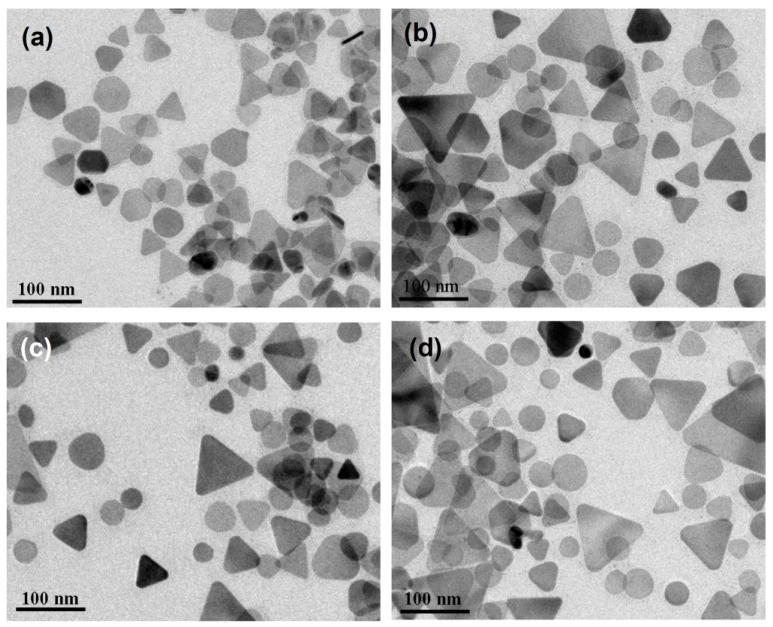
TEM images of the silver nanoparticles synthesized at 60 °C and at different reaction times: (**a**) 35 min; (**b**) 80 min; (**c**) 210 min and (**d**) 300 min.

Figure 6a–d shows the TEM images of the silver colloids synthesized at 35, 80, 210 and 300 min, respectively, at 60 °C. At the early reaction stage, 35 min, almost all the nanostructures are plate-like shapes with edge lengths or diameters equal or smaller than 70 nm. Hardly any nanoparticles with multiple twinned structures can be found at this reaction stage. At 80 min, several nanoprisms with edge length larger than 90 nm can be observed. In the later reaction stage (200 and 300 min), the larger nanoprisms became even larger and the smaller nanoplates became rounder than they were at the early stage. This TEM observation is consistent with the time-dependent extinction spectra shown in Figure 4a.

**Figure 7:**
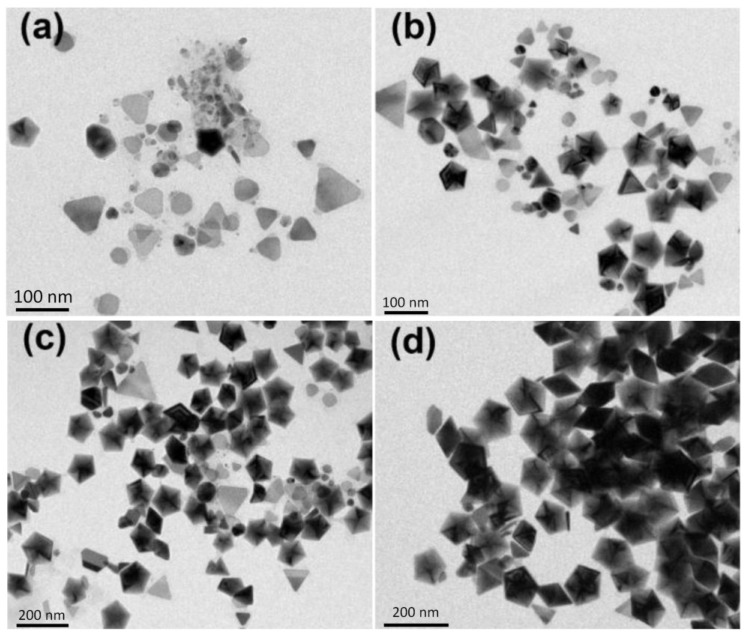
TEM images of the silver nanoparticles synthesized at 10 °C and at different reaction times: (**a**) 7 h; (**b**) 19 h; (**c**) 27 h and (**d**) 45 h.

**Figure 8:**
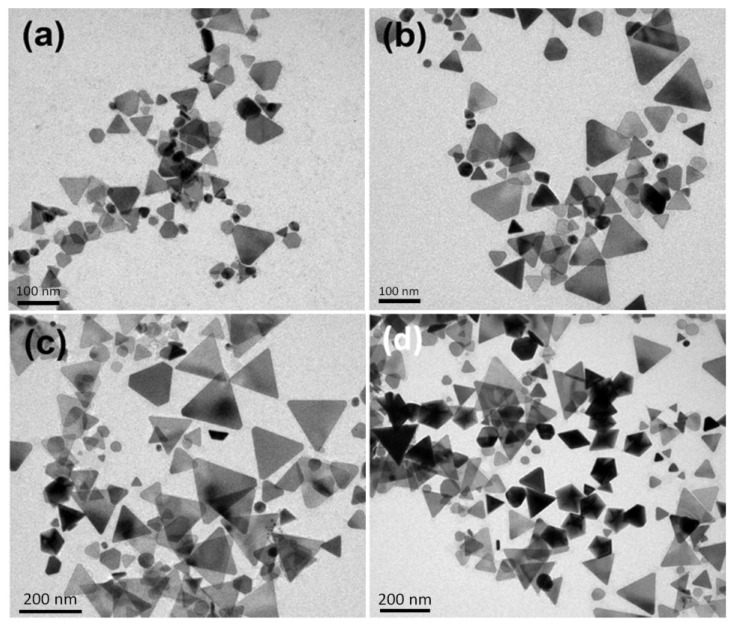
TEM images of the silver nanoparticles synthesized at 20 °C and at different reaction times: (**a**) 4 h; (**b**) 7 h; (**c**) 21 h and (**d**) 25 h.

**Figure 9:**
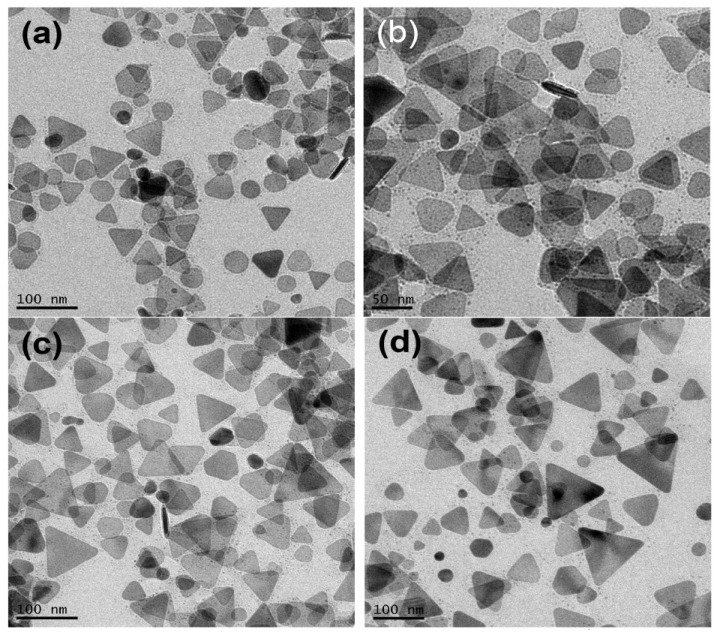
TEM images of the silver nanoparticles synthesized at 40 °C and at different reaction times: (**a**) 90 min; (**b**) 140 min; (**c**) 210 min and (**d**) 11.5 h.

Figure 7, Figure 8 and Figure 9 show the TEM images synthesized at various reaction times at 40, 20 and 10 °C, respectively. From those time-dependent TEM images, we can conclude that the silver nanoplates formed at the early reaction stage at 0–60 °C. According to reference [55], the silver nanoplates were formed in the early stage because the citrate ions can preferentially bind to (111) facets, hence plate-like silver nuclei possess the highest relative stability. The other possibility is the seed-coalescence caused by the light irradiation [35]. When light is irradiated on the silver seeds, the strong local EM field surrounded the particles could cause coalescence of the seeds. Once the primary coalescence is formed, the EM field in the particular plane, which would be stronger than in the perpendicular direction due to the interaction between the dipolar SPR and the light, will cause two-dimensional growth, hence generating the plate-like nanostructures. However, the nanoplates transformed into nanodecahedra when the bath temperature was 0–10 °C and the nanoplates grew into larger nanoprisms or became smaller and rounder caused by the photo-driven etching effect [53] when the bath temperature was 60 °C.

Why does the reaction at a lower temperature lead to nanodecahedra and lead to nanoplates at a higher temperature? It could be possibly explained using the thermodynamic equation, ΔG = ΔH − TΔS. The silver nanodecahedra have a larger particle volume than the silver nanoplates (the volume ratio of nanodecahedra to nanoplates is approximately 10:1), hence possibly they also have a smaller molar entropy and smaller heat of formation (because the average coordination number of silver atoms in nanodecahedra is larger than that in nanoplates). The reaction controlled at a low temperature leads to nanodecahedra because ΔH dominates at a lower temperature (|ΔH| > |TΔS|). However, the reaction controlled at a high temperature leads to silver nanoprisms because becomes the more important factor (|TΔS| > |ΔH|) at a higher temperature.

### 2.3. The Stability of the As-Prepared Silver Nanoparticles

**Figure 10:**
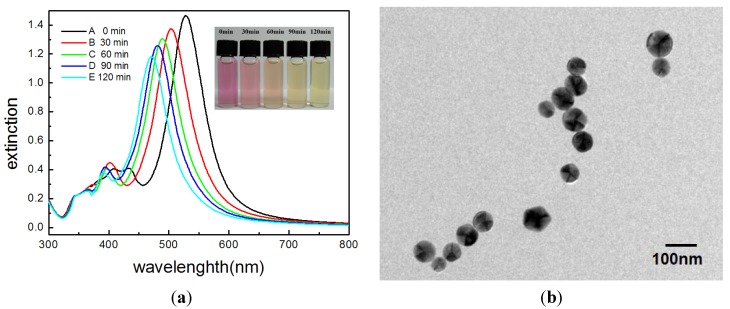

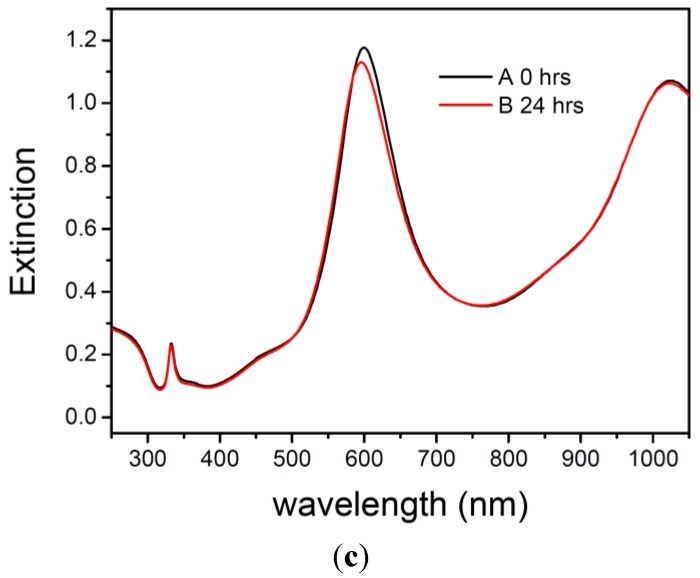
(**a**) Digital photographs and the corresponding UV-vis spectra of decahedral silver nanoparticle (NP) colloids synthesized at 0 °C and held at ambient laboratory conditions: (**A**) 0 min; (**B**) 30 min; (**C**) 60 min; (**D**) 90 min; (**E**) 120 min after the end of the photochemical reaction; (**b**) TEM images of the blunt decahedral silver nanoparticles (without surface protection by 16-MHDA); (**c**) Extinction spectra of silver nanoplates synthesized at 60 °C at 0 h and 24 h after synthesis.

Figure 10a shows the picture and the corresponding time-dependent spectra of the as-prepared silver nanodecahedral colloids (synthesized at 0 °C for 100 h) after removing the excitation light source and placed at ambient temperature for 0, 30 60, 90, and 120 min. The picture shows that the color of the silver colloids changed from pink to orange, and finally to yellow after 120 min. Time-dependent spectra show that the longitudinal SPR band blue-shifted from 530 nm to 460 nm and the transverse SPR mode also blue-shifted from 420 nm to 395 nm. Both the color change and the spectral change are attributed to the change of the morphologies of the silver nanodecahedra.

Figure 10b shows the TEM images of the silver nanodecahedra, without the addition of 16-MHDA for surface protection, in the laboratory environment for 6 h. The two-dimensional projections of these nanostructures are almost circular in shape. Because of that, the corners and edges of the nanodecahedra were seriously etched; however, the distinct character of decahedra, multiple-twinned defect, remained.

On the other hand, Figure 10c shows that extinction spectra of silver colloids synthesized at 60 °C remained almost the same in ambient condition for 24 h. We assumed that the instability of silver nanodecahedra synthesized at such a low temperature resulted from the defects in the crystalline structures. These defects might probably result from the fact that the silver atoms cannot easily rearrange their locations to more suitable sites due to a low average kinetic energy at such a low bath temperature.

### 2.4. SERS Measurements in the Silver NP Colloids

R6G, which has a high fluorescence quantum yield and large Raman scattering cross section, has been used extensively for evaluating the activity of SERS substrates. Nie *et al.* [18] reported that the SERS signal of R6G is strong enough for single-molecule detection. Hence, we also used this probe molecule to compare the enhancement capabilities of several kinds of silver colloids.

**Figure 11:**
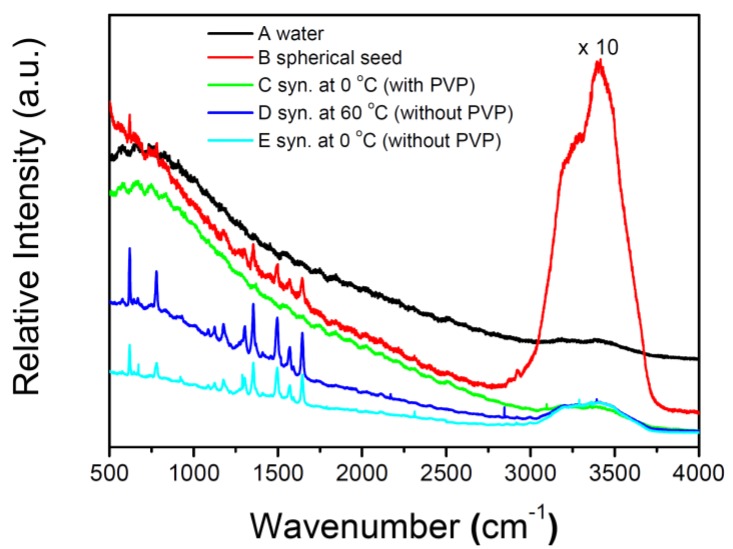
Fluorescence or surface-enhanced Raman spectroscopy (SERS) spectra of 5 × 10^−9^ M R6G probe molecule excited with a 532-nm laser in several kinds of solutions: (**A**) in water; (**B**) spherical silver NP seed colloid; (**C**) decahedral silver NP colloid (synthesized at 0 °C in the presence of Polyvinylpyrrolidone (PVP)); (**D**) silver nanoplate colloid (synthesized at 60 °C); (**E**) decahedral silver NP colloid (synthesized at 0 °C without the addition of PVP and 16-mercaptohexadecanoic acid (16-MHDA)). 0.05 M KBr were added to the above solutions for 30 min prior to spectroscopic measurements.

Figure 11 shows the fluorescence or SERS spectra of R6G in water or several kinds of silver colloids. The intensities of the SERS signals of R6G in as-prepared decahedral silver NP colloids synthesized at 0 °C and in silver nanoplate colloids synthesized at 60 °C are about 16 and 12 times, respectively, stronger than that in the colloids of spherical silver NP seeds. In contrast, the R6G only exhibits a fluorescence background but no prominent Raman signal in the nanodecahedral silver NP colloids synthesized in the presence of PVP using the method developed by Kitaev *et al.* [33] We guessed that PVP molecules in the silver colloids (using the method reported by Kitaev *et al.* [33]) could probably obstruct the adsorption of R6G onto the NP surfaces [52]. In order to confirm our assumption, we added PVP into the as-prepared silver nanodecahedral colloids prior to SERS measurement. It was found that only the fluorescence background but no prominent Raman signal was observed. These results indicate that not only the morphologies of silver NPs but also the reagents for stabilizing can affect the enhancement capability of the colloids. Therefore, the as-prepared silver colloids synthesized by our method, in the absence of PVP and surfactants, would have approachable silver NP surfaces and fewer limitations for SERS applications.

## 3. Experimental Section

### 3.1. Materials

Silver nitrate, sodium citrate, sodium borohydride (NaBH_4_), R6G, KBr, 16-mercaptohexadecanoic (HS(CH_2_)_15_CO_2_H, 16-MHDA) and PVP (Mw = 40,000) were all purchased from Sigma-Aldrich. All these reagents were not further purified before being used. Milli-Q grade water (>18 MΩ) was used in all of the experiments.

### 3.2. Instrumentation

Joel JEM-2100 transmission electron microscopy (TEM) was employed to obtain TEM images of each sample, and was operated at 100 KV and 200 KV. Scanning electron microscopy, SEM, was performed with a Hitachi S-4800 microscope. Before being analyzed by TEM and SEM, the silver colloid was dripped onto a carbon-coated copper grid and air-dried at room temperature. All UV-vis extinction spectra were recorded at 25 °C on a Hitachi U-2800 spectrophotometer using a quartz cuvette with an optical path of 10 mm.

### 3.3. Colloid Preparation

**Figure 12:**
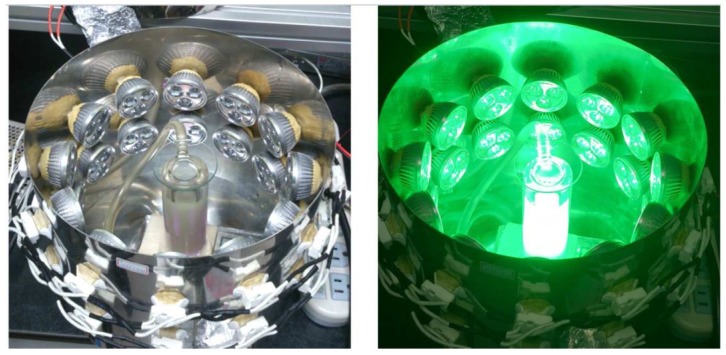
The setup for the temperature-controlled photochemical reaction with green LEDs.

The spherical silver seeds were prepared by the procedures described previously [16]. Briefly, 1 mL of sodium citrate (3.0 × 10^−1^ M) and 1 mL of silver nitrate (1.0 × 10^−2^ M) were mixed with 97.8 mL pure water with rapid stirring. The mixture was then added dropwise with 0.2 mL sodium borohydride (1.0 × 10^−2^ M) under vigorous magnetic stirring. The solution immediately turned yellow, an indication of formation of spherical silver colloids. After being stirred for a further 30 min, the spherical silver seed colloids were then incubated in a temperature-controlled container at 0, 5, 10, 20, 40, and 60 °C, respectively, and were irradiated with the green LEDs (*λ*_max_ = 520 ± 18 nm, average power ≈25 mW/cm^2^). The setup for photochemical reaction is similar to our previous study and shown as Figure 12 [53]. The irradiation time needed to convert the spherical nanoparticles into nanostructures with other shapes is temperature dependent. We stopped the reaction when the peak intensities of the time-dependent SPR spectra reached the maxima. The reactions times were 5, 11.5, 25, 45, 72, and 100 h when the bath temperatures were set at 60, 40, 20, 10, 5, and 0 °C, respectively.

### 3.4. SERS Measurement

A solution of R6G (10^−8^ M, 0.9 mL) was added to 1 mL of silver colloids (four kinds: spherical seeds, silver nanoplates synthesized at 60 °C, decahedral silver nanoparticles synthesized at 0 °C, and decahedral silver nanoparticles synthesized at 0 °C in the presence of 7 × 10^−4^ M PVP). After being stirred at room temperature for 30 min, the mixtures were added with KBr (1 M, 0.1 mL). Prepared samples were placed in a quartz cuvette, and excited by a 30 mW 532 nm laser beam (Coherent, DPSS 532). The typical acquisition time of a SERS spectrum was 40 s.

## 4. Conclusions

To summarize, we synthesized silver NPs by plasmon-mediated shape transformation from spherical NPs to nanostructures of other shapes under irradiation of green LEDs at various temperatures. We found that temperature is a very important factor in controlling the morphology of NPs, *i.e.*, the major products are silver nanoplates at 60 °C but became decahedral silver NPs at 0 °C. This result can be explained using the concept of thermodynamics. Additionally, it was found that the tips and edges of silver decahedral NPs became slowly blunt at ambient temperature while the silver nanoplates were robust in retaining their morphology for a quite long time. The instability of silver nanodecahedra might probably result from defects in the nanostructures due to the fact that the silver atoms cannot rearrange their occupied positions easily because of the low average kinetic energy at such a low bath temperature, 0 °C. Finally, we showed that both silver nanoplate colloids synthesized at 60 °C and decahedral silver NP colloids synthesized at 0 °C in the absence of PVP exhibited good SERS activities for R6G.
